# An Analysis of the Mineral Composition of Pink Salt Available in Australia

**DOI:** 10.3390/foods9101490

**Published:** 2020-10-19

**Authors:** Flavia Fayet-Moore, Cinthya Wibisono, Prudence Carr, Emily Duve, Peter Petocz, Graham Lancaster, Joanna McMillan, Skye Marshall, Michelle Blumfield

**Affiliations:** 1 Nutrition Research Australia, Level 10, 20 Martin Place, Sydney 2000, Australia; cinthya@nraus.com (C.W.); prue@nraus.com (P.C.); emily@nraus.com (E.D.); skye@nraus.com (S.M.); michelle@nraus.com (M.B.); 2 Department of Statistics, Macquarie University, Sydney 2109, Australia; peter.petocz@mq.edu.au; 3 Environmental Analysis Laboratory, Southern Cross University, Lismore 2480, Australia; graham.lancaster@scu.edu.au; 4 Department of Dietetics, Nutrition and Sport, School of Allied Health, Human Services and Sport, La Trobe University, Bundoora Victoria 3086, Australia; jo@drjoanna.com.au; 5 Bond University Nutrition & Dietetics Research Group, Faculty of Health Sciences & Medicine, Bond University, Gold Coast, QLD 4226, Australia

**Keywords:** pink salt, salts, sodium chloride, minerals, heavy metals, lead poisoning

## Abstract

Little is known about the mineral composition of pink salt. The aim of this study was to evaluate for the first time the mineral composition of pink salt available for purchase in Australia and its implications for public health. Pink salt samples were purchased from retail outlets in two metropolitan Australian cities and one regional town. Color intensity, salt form, and country of origin were coded. A mass spectrometry scan in solids was used to determine the amount of 25 nutrients and non-nutritive minerals in pink salt (*n* = 31) and an iodized white table salt control (*n* = 1). A wide variation in the type and range of nutrients and non-nutritive minerals across pink salt samples were observed. One pink salt sample contained a level of lead (>2 mg/kg) that exceeded the national maximum contaminant level set by Food Standards Australia New Zealand. Pink salt in flake form, pink salt originating from the Himalayas, and darker colored pink salt were generally found to contain higher levels of minerals (*p* < 0.05). Despite pink salt containing nutrients, >30 g per day (approximately 6 teaspoons) would be required to make any meaningful contribution to nutrient intake, a level that would provide excessive sodium and potential harmful effects. The risk to public health from potentially harmful non-nutritive minerals should be addressed by Australian food regulations. Pink salt consumption should not exceed the nutrient reference values for Australia and New Zealand guidelines of <5 g of salt per day.

## 1. Introduction

Salt is composed of sodium chloride and occurs naturally in many foods including milk, eggs, and shellfish [1]. The availability of salt has been essential to civilization through its use as a flavor enhancer and as a preservative in food products [2]. Historically, salt was produced by boiling brine sourced from a variety of natural sources including seawater, wells, lakes, and salt springs, and it can also be found as rock salt in mines [3,4].

Sodium is an element found in salt and is deemed necessary for certain human physiological functions including the regulation of extracellular fluid volume, nerve conduction, and muscle function [2,5,6]. Salt is now widespread in packaged foods and most adults easily exceed the World Health Organization’s (WHO) recommendation of less than 2 g of sodium per day (equivalent to approximately 5 g of salt per day) [7,8]. In Australia, the same recommendation is provided in the nutrient reference values (NRV) for Australia and New Zealand as a suggested dietary target (SDT) for adults, set to prevent chronic disease [5]. Based on data from the Australian Health Survey (AHS) 2011–2012, the average sodium intake for Australians was 2404 mg per day [9], but actual intake is likely to be much higher (i.e., approximately 3600 mg per day) as salt added at the table or in food preparation at home was excluded from this analysis [9].

Worldwide there is a wide variety of salt, beyond white table salt, available for purchase. In recent years, pink salt, particularly pink Himalayan salt, has grown in popularity with increased media attention [10]. It is often marketed for its alleged health benefits and is positioned to be nutritionally superior to white table salt [11,12,13,14]. Few studies have reported the mineral content of pink salts internationally [4,15,16], and found pink salt to contain a variety of essential nutrients including iron, zinc, and calcium, but found some samples also contained impurities or relatively large amounts of non-nutritive minerals such as arsenic, lead, and cadmium [15]. No study has evaluated the nutritional composition of pink salt available for purchase. Non-nutritive minerals such as arsenic, cadmium, lead, or mercury have no established health benefit and in relatively small doses, lead to multiple organ damage [16,17,18,19]. Given the increased consumer interest in pink salt and the potential risk of harmful non-nutritive mineral contamination, an investigation into the mineral composition of pink salt in Australia is warranted. The aim of this study was to evaluate, for the first time, the mineral composition of pink salt available for purchase in Australia and to determine its implications for public health.

## 2. Materials and Methods 

### 2.1. Sample Collection

Every pink salt sample commercially available was purchased from four major supermarkets, green grocers, and health food shops in two metropolitan Australian cities and one regional town (Sydney, NSW, Australia; Canberra, ACT, Australia; Coffs Harbour, NSW, Australia) in June 2018. If available, different forms of pink salt within the same brand were purchased. These included table or finely ground, flakes, and coarse/rock (grinder) salt. An iodized fine white table salt was purchased as the control sample, as this is the salt currently mandated for iodine fortification of commercial bread products by Food Standards Australia New Zealand (FSANZ) [20]. Each salt’s region of origin and pricing information were also recorded. Pricing information was collected from online retail stores on the 18th December 2019. For items with more than one price and size option, pricing for the smaller size option was chosen as it was assumed that consumers do not habitually purchase salt in bulk volumes. The price per 100 g was then calculated.

### 2.2. Sample Analyses

Each pink salt sample was transferred from the original packaging into clear plastic bags, and randomly numbered and labelled from two to 32, with the control sample numbered as one. De-identified pink salt samples were coded according to their color intensity. Three researchers (CW, ED, SM) independently coded each sample’s color intensity (0 = no color, 1 = light, 2 = medium, 3 = dark pink), and in the case of a discrepancy, samples were re-coded until a consensus was achieved. 

The control and the pink salt samples were sent to the National Association of Testing Authorities, Australia (NATA) accredited Environmental Analysis Laboratory (EAL) at Southern Cross University for analysis. A mass spectrometry scan in solids was used to determine the amount (mg/kg) of minerals (aluminum, arsenic, barium, boron, cadmium, calcium, chromium, cobalt, copper, iron, lead, magnesium, manganese, mercury, molybdenum, nickel, phosphorus, potassium, selenium, silicon, silver, sodium, sulfur, vanadium, zinc) in a single test for each salt sample. To do this, approximately 0.4 g for each sample was digested in an Aqua Regia matrix (2.5 mL HNO3: 7.5 mL HCl) on a hot block at 120 degrees for 1 h. Once digestion was complete the samples were analyzed on a PerkinElmer (Waltham, MA, USA) NexION 300D inductively coupled plasma mass spectrometer (ICPMS). The ICPMS is capable of operating in three modes of analysis i.e., standard, kinetic energy discrimination (KED), and dynamic reaction cell (DRC), enabling interference removal and superior detection limits in difficult matrices.

### 2.3. Mineral Classification

The 25 minerals were classified as either nutrients or non-nutritive minerals based on the NRV [5]. Minerals with an NRV were classified as nutrients, and those without an NRV as non-nutritive minerals. The most appropriate NRV for comparison with individual intake is the recommended dietary intake (RDI), adequate intake (AI), and upper level (UL) of intake [5]. The RDI is the daily nutrient level estimated to meet the requirements of nearly all (97 to 98%) healthy individuals in a particular life stage and sex group [5]. When an RDI was not able to be set, an AI, which is the average daily nutrient intake level that is assumed to be adequate, is used instead [5]. The UL is the highest average daily nutrient level that is likely to pose no adverse effects to individuals. When an NRV, e.g., a UL, was not available for a nutrient, other international guidelines were used from FSANZ [21], the Codex Alimentarius Commission [22], the Institute of Medicine [23], and research literature [24]. The nutrient composition of 5 g (approximately one teaspoon) of pink salt, representing the maximum recommended intake by the Australian Dietary Guidelines [25] and the WHO [7], were compared to the NRV.

### 2.4. Statistical Analyses

The composition of nutrients and non-nutritive minerals for all samples was analyzed using descriptive statistics. A one-way analyses of variance (ANOVA) was conducted to identify if mineral content differed by form (finely ground, flakes, or coarse form) or color intensity (no color, light pink, medium pink, or dark pink). A two-sample *t*-test was conducted to determine if there were any differences in mineral content by region of origin (Himalayas vs. other). A one sample *t*-test was conducted to confirm if there were differences in mineral content between pink salt and the white table salt control. Analyses were performed using Minitab 17 and SAS University Edition. *p*-values < 0.05 were considered statistically significant.

## 3. Results

The description of pink salt samples (*n* = 31) and iodized white table salt (*n* = 1) available in Australia are presented in Table 1. The majority of pink salts were labelled “Himalayan” (*n* = 27, 87%), with others labelled as “pink” but not “Himalayan” (*n* = 3, 10%), or unbranded and described as “Australian” (*n* = 1, 3%). The region of origin included the Himalayas (*n* = 27, 87%), Australia (*n* = 3, 10%), or Peru (*n* = 1, 3%). Pink salts were sold in three different forms that included coarse or grinder form (*n* = 15, 48%), table salt or fine form (*n* = 14, 45%), and flakes (*n* = 2, 7%). The color intensity of pink salt ranged from dark (*n* = 13, 42%), medium (*n* = 11, 36%), light (*n* = 5, 16%), and no color (*n* = 3, 6%).

### 3.1. Mineral Composition

The nutrient and non-nutritive mineral composition of pink salt and white table salt is summarized in Table 2, Table 3, respectively. Large variations in values were observed for calcium (530.62–5736.73 mg/kg), magnesium (146.78–11937.98 mg/kg), potassium (98.39–4528.89 mg/kg), iron (0–167.52 mg/kg), and sodium (343,384.79–459,115.58 mg/kg). Copper, chromium, and manganese were found in very small amounts (i.e., <1.00 mg/kg) (Table 2). Few samples contained molybdenum (*n* = 8; <0.15 mg/kg), phosphorus (*n* = 3; <28.90 mg/kg), selenium (*n* = 6; <0.19 mg/kg), and zinc (*n* = 3; <0.86 mg/kg). Large variation in values were reported for the non-nutritive mineral’s aluminum (0–192.65 mg/kg), lead (0–2.59 mg/kg), silicon (0–372.10 mg/kg), and sulfur (1703.97–33,754.34 mg/kg) (Table 3). Neither arsenic nor silver were detected in any of the pink salt samples, and vanadium was detected in one sample (0.12 mg/kg). Very low amounts of barium, boron, cadmium, cobalt, mercury, and nickel were detected in some of the pink salt samples. 

Compared to white table salt per kilogram, pink salt contained substantially higher nutrient levels of calcium (2695.09 mg vs. 393.28 mg; *p* < 0.001), iron (63.75 mg vs. 0 mg; *p* < 0.001), magnesium (2655.31 mg vs. 83.94 mg; *p* < 0.001), manganese (2.16 mg vs. 0 mg; *p* < 0.001), and potassium (2406.05 mg vs. 151.68 mg; *p* < 0.001), and lower levels of sodium (394,717.96 mg vs. 427,636.27 mg; *p* < 0.01) (Table 4). Compared to white table salt per kilogram, pink salt contained higher non-nutrient mineral levels of aluminum (76.27 mg vs. 0 mg; *p* < 0.001), barium (0.77 mg vs. 0.01 mg; *p* < 0.001), silicon (131.00 mg vs. 0 mg; *p* < 0.001), and sulfur (7344.70 mg vs. 431.22 mg; *p* < 0.001) (Table 4). 

When compared to nutrient recommendations (Table 4), one pink salt sample, which was the only sample from Peru, contained a high lead content (2.59 mg/kg) which exceeded the maximum metal contaminant level of 2 mg/kg for salt [26]. No other pink salt sample exceeded the maximum level (mg/kg) for metal contaminants (arsenic, cadmium, or mercury) or the UL set by FSANZ and the NRV, respectively. The mean dietary intake of nutrients and non-nutritive minerals in 5 g of pink salt (i.e., one teaspoon) are provided in Table 4. All nutrient levels were well below the RDI or AI, with the exception of sodium which met the SDT of 2000 mg/day, similarly to white table salt. 

### 3.2. Origin 

Mean differences in nutrients according to region (i.e., Himalayas vs. other regions) were found for copper (0.08 mg/kg; *p* = 0.045), iron (54.95 mg/kg; *p* = 0.026), potassium (2507.87 mg/kg; *p* < 0.001), and zinc (−0.41 mg/kg; *p* < 0.001) (Table 5). Differences in non-nutritive minerals were found for aluminum (82.99 mg/kg; *p* = 0.002), barium (0.83 mg/kg; *p* < 0.001), cadmium (−0.01 mg/kg; *p* = 0.008), cobalt (0.4 mg/kg; *p* = 0.007), lead (−0.6 mg/kg; *p* = 0.011), silicon (150.41 mg/kg; *p* = 0.002), and sulfur (−6186.04 mg/kg; *p* = 0.030). A positive mean difference indicates a higher nutrient level in samples of Himalayan origin, while a negative mean difference indicates a higher nutrient level in samples from other regions. Higher values were mostly attributed to pink salt originating from the Himalayas, with the exception of zinc, cadmium, lead, and sulfur which were lower (Table 5).

### 3.3. Form

Copper, manganese, sodium, and cadmium levels were higher in flakes compared to finely ground pink salt (mean differences: copper 0.15 mg/kg, *p* < 0.05; manganese 3.17 mg/kg, *p* < 0.05; sodium 60,998.00 mg/kg, *p* < 0.05; cadmium 0.01 mg/kg, *p* < 0.05) and coarse samples (mean differences: copper 0.17 mg/kg, *p* < 0.05; manganese 2.80 mg/kg, *p* < 0.05; sodium 64,374.00 mg/kg, *p* < 0.05; cadmium 0.01 mg/kg, *p* < 0.05) (Table 5). Potassium was higher in coarse samples compared to those finely ground (mean difference: 1171.70 mg/kg, *p* < 0.05). Nickle was higher in flakes compared to coarse pink salt samples (mean difference: 0.20 mg/kg, *p* < 0.05)

### 3.4. Color Intensity 

Dark pink samples contained higher levels of calcium (mean differences: 2583.90 mg/kg, *p* < 0.05), potassium (2747.40 mg/k, *p* < 0.05), aluminum (98.47 mg/kg, *p* < 0.05), barium (0.97 mg/kg, *p* < 0.05), and silicon (179.63 mg/kg, *p* < 0.05), and lower levels of magnesium (−5528.70 mg/kg, *p* < 0.05) and boron (−1.57 mg/kg, *p* < 0.05), compared to no color samples (Table 5). Similar trends were found when dark pink samples were compared to light pink samples; whereas medium pink samples contained higher levels of potassium (mean differences: 2214.40 mg/kg, *p* < 0.05) and barium (0.82 mg/kg, *p* < 0.05), and lower levels of magnesium (−5881.60 mg/kg, *p* < 0.05), boron (−1.76 mg/kg, *p* < 0.05), and sulfur (−16,688.00 mg/kg, *p* < 0.05) compared to no color samples. 

## 4. Discussion

This is the first study to analyze the mineral composition of pink salt available for purchase in Australia, and to compare it to an iodized white table salt control. Pink salt contained substantially higher levels of calcium, iron, magnesium, manganese, potassium, aluminum, barium, silicon, and sulfur, but lower levels of sodium compared to the white table salt. One teaspoon (5 g) of pink salt contained small quantities of minerals but did not make a clinically significant contribution to nutrient intake, as levels were too low in comparison to the NRVs, with the exception of sodium, which reached the SDT. All samples met the FSANZ safe level of metal contaminants or the UL set by the NRV, with the exception of one sample, which exceeded the maximum contaminant level for lead, posing concerns for public health. Very wide variations in the type and range of nutrients and non-nutritive minerals were found in pink salt, with those in flake form, of Himalayan origin and darker color generally found to contain higher levels of minerals.

Despite the many nutrients found in pink salt that are essential for health, (e.g., calcium, iron, magnesium, and potassium), an exceedingly high intake of pink salt (>30 g per day) would be required before this made any clinically significant contribution to nutrient intake [7]. Not only is such an amount unrealistic in usual diets, at 30 g the SDT and WHO recommendations for sodium would be exceeded by 592% [5]. There is strong evidence that a diet high in sodium is associated with hypertension, a major risk factor for the development and progression of cardiovascular disease and several other diseases including stroke, kidney disease, and stomach cancer [27,28,29,30,31,32]. Sodium intake among Australian adults is reported between 2400 mg to 3600 mg per day [9]; however, intake is likely to be higher as this value is derived from sodium naturally occurring in foods or added during the manufacturing process, and excludes that from salt added at the table. To achieve a sodium intake which does not exceed the SDT, the Australian Dietary Guidelines recommend that no salt is added during food preparation process or at the table [25]. In this study, both pink salt and white table salt easily reached the SDT for sodium, despite pink salt having a slightly lower sodium content. The difference between pink and white salt was for non-sodium nutrients, but the level was not clinically relevant, especially given the high amount of sodium that is ingested with these nutrients. Given there is no room in the Australian dietary intake for pink or white salt to be added to cooking or meals, nutrients should instead be obtained from low-sodium and high nutrient foods such as fruits, vegetables, lean meats and their alternatives, legumes, nuts, seeds, and cereal (grain) foods [25].

While the links between high sodium intake and adverse cardiovascular health are well known, a J-shaped relationship between sodium intake and mortality has been recently reported [33,34,35]. Research suggests that humans could be genetically programmed with a ‘personal index of salt sensitivity,’ which determines the amount of daily salt consumption that will have the least negative impact on their health [36]. It is estimated that 11 to 16 percent of individuals are inverse salt sensitive and need to consume a higher amount of sodium to prevent high blood pressure [36,37]. Some individuals require up to 10 g of salt per day (2 teaspoons) to lower blood pressure into the range considered safe by blood pressure guidelines (<120/<80 mm Hg) [36,38,39]. While it is acknowledged that not all individuals will benefit from a low salt diet, it is possible the varying levels of toxic heavy metals found in pink salt may contribute to poor health outcomes even in those individuals with inverse salt sensitivity.

Reasons for the high variability in mineral content between pink salt samples are poorly understood and could be due to the soil and rock profile and quality where the salt was harvested. Variations in soil quality between countries have been shown to influence differences in the non-nutritive mineral content of pink salt [4]. In a recent study that examined different types of salts, including pink Himalayan salt, higher concentrations of aluminum, silicon, potassium, titanium, magnesium, and iron were found in pink Himalayan salts, compared to salts that originated from Brazil [4]. Similarly, our findings show that the nutrient composition of pink salt differs by region of origin, where pink salt from the Himalayas reported higher amounts of iron, aluminum, silicon, cobalt, barium, and potassium, compared to other regions; and lower lead content compared to Peru. Darker colored samples were also found to contain higher mineral levels, compared to other samples. The concentrated shade of pink in pink salt is determined by traces of iron oxide (i.e., rust) or impurities in the soil [4]. While the absorption of iron oxide is similar to iron sulfate and in high doses could be useful as an iron supplement [40], no differences in iron content by color intensity were observed in this study.

Poorly planned urbanization, mining, industrial processing, and heavy use of metal-based chemicals are some activities recognized to cause contamination of a country’s food and water supply [41,42,43,44]. In Pakistan, which produces Himalayan pink salt for commercial distribution, an increase in industrialization and population expansion into urban areas has led to environmental pollution, causing soil and water contamination [43]. Potentially toxic non-nutritive minerals such as cadmium have been detected in Pakistani soil, leading to further contamination of the country’s food and water supply [43]. In this study, traces of cadmium were detected in pink salt samples originating from the Himalayas, and in an Australian pink salt sample. While cadmium levels did not exceed the FSANZ maximum contaminant levels, anthropogenic contamination in Australia is less common relative to some South East Asian cities [45] or European cities [46]. This may be attributed to differences in the treatment of industrial wastewater and agricultural practices between countries [45]. Although Australia does not face the same level of issues of over-population or rapid urbanization as other countries, it still is prudent to be aware of unintentional exposure to non-nutritive mineral contaminants introduced through the food supply, especially when the food is being perceived as nutritious and to confer a health benefit [47].

The single pink salt sample from Peru contained a very high level of lead which exceeded the FSANZ maximum contaminant level (2.59 mg/kg vs. 2 mg/kg) and had 130 times more lead than the iodized white table salt control. Non-nutritive minerals such as lead are not biodegradable and can therefore cause harmful effects on human health when consumed through food. There is no level of exposure to lead that is known to be without harmful effects. These effects may present as acute or chronic symptoms including compromised bone health, gastrointestinal discomfort, respiratory distress, kidney dysfunction, cognitive decline, heart problems, or even cancer [17,18,41,42,48]. Although the mineral content in this sample was only measured once, Peru ranks among the world’s top five producers of lead and the WHO has confirmed lead exposure from soil in Peru [49]. It is of great concern that there is pink salt sold in Australia that exceeds the maximum level of lead permitted. Further research and greater regulatory control are required to ensure that pink salt available in Australia is safe for consumption.

Limitations of this study include potential selection bias through the inclusion of 31 pink salt samples from two metropolitan Australian cities and one regional town. While researchers attempted to purchase all available pink salt samples to create a representative sample, additional pink salt products may be available in other parts of Australia. Results for the difference in mineral content by form should be interpreted with caution due to an increased risk of type 2 error, a consequence of a small sample size of flake samples (*n* = 2). Although color coding was completed by three researchers independently, a subjective measure of the color intensity was used and there was potential for misclassification bias. Lastly, nutrient composition was measured once by one laboratory which could lead to measurement bias. Findings are strengthened by two different versions of the same branded product being included where available. This is the first study to provide insight into the mineral composition of pink salt available in Australia and thus represents an important contribution to the literature. 

## 5. Conclusions

This study provides evidence that there are both nutritive and non-nutritive minerals in pink salt available for purchase in Australia, but that wide variations exist and there is the potential for contaminant ingestion. While one teaspoon (5 g) of pink salt contained small quantities of all nutrients, the levels did not meaningfully contribute to nutrient intake, with the exception of sodium which reached the Australian suggested dietary target. Any potential health benefits provided by the higher nutrient content in pink salt would be counteracted by the large amount of sodium that would also be consumed. Importantly, one pink salt sample from Peru contained a level of lead which exceeded the FSANZ maximum contaminant level. The risk to public health from potentially harmful non-nutritive minerals needs to be addressed by further research and further investigated by food regulatory bodies. Pink salt should only be consumed according to Australian guidelines at <6 g of salt per day from all food and beverage sources.

## Figures and Tables

**Table 1:** Description of pink salt (*n* = 31) and iodized white table salt (*n* = 1) samples purchased from retail stores in Australia in June 2018.

Brand	Product Name	Form	Origin	Color Intensity	Price ($) ^1^ per 100 g
**Control**					
SAXA	Iodized white table salt	Fine	N/A	No color	1.60 ^2^
**Pink salt**					
Affordable wholefoods	Pink Himalayan Salt	Fine	Himalayan	Light pink	1.19 ^3^
Chef’s choice	Himalayan Pink Fine Salt	Fine	Himalayan	Medium pink	1.76 ^4^
Chef’s choice	Himalayan Pink Rock Salt with Grinder	Coarse	Himalayan	Dark pink	1.67 ^5^
Coles	Himalayan Pink Salt Grinder	Coarse	Himalayan	Dark pink	2.91 ^6^
Go Vita	Himalayan Salt	Fine	Himalayan	Medium pink	1.19 ^7^
Hoyt’s	Pink Himalayan Rock Salt	Coarse	Himalayan	Dark pink	1.30 ^5^
Lotus	Himalayan Salt Coarse	Coarse	Himalayan	Dark pink	1.16 ^8^
Masterfoods	Pink Himalayan Salt	Coarse	Himalayan	Dark pink	7.77 ^9^
McKenzie’s	Himalayan Fine Pink Salt	Fine	Himalayan	Medium pink	1.32 ^5^
McKenzie’s	Himalayan Pink Rock Salt	Coarse	Himalayan	Dark pink	1.25 ^2^
Mount Zero	Pink lake natural salt	Coarse	Australian	No color	6.99 ^10^
Murray River Gourmet	Salt Flakes Naturally Pink	Flakes	Australian	Light pink	5.00 ^10^
*n* foods	Himalayan Rock Sat	Coarse	Himalayan	Dark pink	-
Nirvana organics	Himalayan Crystal Salt Medium	Fine	Himalayan	Dark pink	2.64 ^11^
Nirvana organics	Himalayan Crystal Salt Granules	Coarse	Himalayan	Dark pink	6.00 ^11^
Nirvana organics	Himalayan Crystal Salt Fine	Fine	Himalayan	Medium pink	2.70 ^12^
Peruvian Pink Salt	Fine Grain Salt	Fine	Peru	Light pink	1.66 ^10^
Real Food That Loves You Back	Himalayan Pink Rock Salt-Fine	Fine	Himalayan	Medium pink	1.16 ^13^
Real Food That Loves You Back	Himalayan Rock Salt Crystals	Coarse	Himalayan	Dark pink	1.16 ^13^
SAXA	Natural Pink Himalayan Table Salt	Fine	Himalayan	Medium pink	2.39 ^2^
SAXA	Pink Himalayan Salt Flakes	Flakes	Himalayan	Medium pink	6.32 ^5^
SAXA	Natural Pink Himalayan Rock Salt	Coarse	Himalayan	Dark pink	0.80 ^2^
SAXA	Natural Pink Himalayan Salt Grinder	Coarse	Himalayan	Dark pink	4.21 ^2^
SHERU	Fine Himalayan Salt	Fine	Himalayan	Medium pink	1.16 ^14^
The gluten free co.	Himalayan Salt Fine	Fine	Himalayan	Light pink	1.90 ^15^
The gluten free co.	Himalayan Salt Coarse	Coarse	Himalayan	Dark pink	1.91 ^15^
Unbranded	Australian Pink Lake Salt (Medium)	Fine	Australian	No color	-
Unbranded	Fine Himalayan Salt	Fine	Himalayan	Light pink	-
Unbranded	Rock Himalayan Salt	Coarse	Himalayan	Medium pink	-
Woolworths	Himalayan Pink Fine Table Salt	Fine	Himalayan	Medium pink	0.53 ^2^
Woolworths	Pink Himalayan Salt	Coarse	Himalayan	Medium pink	2.73 ^2^

^1^ Prices (in Australian dollars) sourced online as at 18 December 2019; ^2^ www.woolworths.com.au; ^3^ www.affordablewholefoods.com.au; ^4^ www.harrisfarm.com.au; ^5^ www.choice.com.au/food-and-drink/groceries/herbs-and-spices/articles/accessible-text-files/salt-prices-compared; ^6^ www.coles.com.au; ^7^ www.govita.com.au; ^8^ www.lotuspantry.com.au; ^9^ www.myozbox.com.au; ^10^ www.doorsteporganics.com.au; ^11^ www.nirvanahealthproducts.com; ^12^ www.naturalhealthorganics.com.au; ^13^ www.goodness.com.au; ^14^ www.nourishedearth.com.au; ^15^ www.meed.com.au; ‘-“represents items with no available pricing information.

**Table 2:** Composition of nutrients in pink salt (*n* = 31) and iodized white table salt (*n* = 1) samples which were purchased from retail stores in Australia in June 2018.

		Nutrient Content (mg/kg)											
Brand	Product Name	Calcium	Chromium	Copper	Iron	Magnesium	Manganese	Molybdenum	Phosphorus	Potassium	Selenium	Sodium	Zinc
**Control**													
SAXA	Iodized white table salt	393.28	0.00	0.09	0.00	83.94	0.00	0.04	0.00	151.68	0.00	427,636.27	0.00
**Pink salt**													
Affordable wholefoods	Pink Himalayan Salt	1799.40	0.09	0.09	44.00	1345.12	1.27	0.00	28.90	2085.71	0.00	394,315.32	0.00
Chef’s choice	Himalayan Pink Fine Salt	3111.17	0.04	0.20	20.10	2499.69	1.24	0.00	0.00	3515.59	0.03	389,074.55	0.00
Chef’s choice	Himalayan Pink Rock Salt with Grinder	3755.33	0.01	0.09	22.99	2550.59	1.30	0.00	0.00	4528.89	0.00	381,948.05	0.00
Coles	Himalayan Pink Salt Grinder	2595.03	0.15	0.08	79.75	2565.36	2.57	0.00	0.00	2505.65	0.00	384,301.64	0.00
The gluten free co.	Himalayan Salt Coarse	3388.74	0.06	0.05	78.99	2529.64	2.09	0.04	0.00	3062.06	0.00	426,467.55	0.00
Go Vita	Himalayan Salt	2426.31	0.26	0.15	167.52	3355.49	3.80	0.00	0.00	2394.43	0.14	381,218.17	0.00
Hoyt’s	Pink Himalayan Rock Salt	3629.45	0.26	0.12	129.66	3415.42	3.26	0.00	0.00	3306.94	0.00	393,437.27	0.00
Lotus	Himalayan Salt Coarse	2915.44	0.18	0.10	103.13	2828.15	2.59	0.02	0.00	2873.87	0.00	387,244.33	0.00
Masterfoods	Pink Himalayan Salt	3269.52	0.08	0.10	44.92	2401.10	1.79	0.00	0.00	3299.52	0.18	384,989.53	0.00
McKenzie’s	Himalayan Fine Pink Salt	2250.53	0.10	0.05	41.80	1700.59	1.95	0.00	0.00	1433.82	0.16	399,641.42	0.00
Mount Zero	Pink lake natural salt	530.62	0.00	0.01	0.00	4454.23	0.36	0.08	0.00	312.08	0.00	380,632.11	0.00
Murray River Gourmet	Salt Flakes Naturally Pink	897.89	0.44	0.10	44.16	990.57	8.61	0.06	0.00	143.31	0.19	449,913.48	0.86
*n* foods	Himalayan Rock Sat	2840.44	0.14	0.11	85.46	2621.64	2.42	0.00	0.00	2572.03	0.00	393,311.56	0.00
Nirvana organics	Himalayan Crystal Salt Medium	2843.62	0.08	0.13	45.69	2309.57	1.83	0.00	0.00	2395.69	0.00	398,812.10	0.00
Nirvana organics	Himalayan Crystal Salt Granules	4421.44	0.16	0.08	70.73	2743.52	2.41	0.00	0.00	3841.33	0.00	402,301.46	0.00
Nirvana organics	Himalayan Crystal Salt Fine	1968.95	0.17	0.08	102.56	2557.86	2.57	0.00	0.00	1808.16	0.00	386,473.56	0.00
Peruvian Pink Salt	Fine Grain Salt	5736.73	0.03	0.02	16.40	146.78	1.31	0.00	0.00	98.39	0.00	387,547.54	0.82
Real Food That Loves You Back	Himalayan Pink Rock Salt-Fine	2262.89	0.12	0.11	88.97	2467.40	2.56	0.00	0.00	2327.98	0.00	401,488.19	0.00
Real Food That Loves You Back	Himalayan Rock Salt Crystals	3398.81	0.28	0.10	155.65	3979.51	3.40	0.00	0.00	3945.90	0.00	393,233.37	0.00
SAXA	Natural Pink Himalayan Table Salt	2966.99	0.61	0.22	54.00	2005.48	1.65	0.05	0.00	3189.23	0.00	413,789.82	0.00
SAXA	Pink Himalayan Salt Flakes	1717.05	0.47	0.41	23.65	918.43	1.17	0.00	0.00	791.40	0.00	459,115.58	0.00
SAXA	Natural Pink Himalayan Rock Salt	3579.58	0.39	0.12	137.26	3259.21	3.42	0.00	0.00	2832.21	0.00	376,824.50	0.00
SAXA	Natural Pink Himalayan Salt Grinder	1268.72	0.00	0.05	0.00	958.44	0.33	0.00	0.00	1599.79	0.00	393,129.90	0.00
SHERU	Fine Himalayan Salt	2273.53	0.13	0.10	65.50	1824.92	1.93	0.00	0.00	1624.70	0.00	394,087.94	0.06
The gluten free co.	Himalayan Salt Fine	1448.13	0.00	0.15	2.62	1184.53	0.74	0.00	0.00	1811.63	0.15	378,099.46	0.00
Unbranded	Australian Pink Lake Salt	768.78	0.00	0.03	3.02	11,937.98	0.52	0.02	7.99	333.36	0.00	343,384.79	0.00
Unbranded	Fine Himalayan Salt	2315.49	0.10	0.08	81.03	2119.48	2.18	0.15	3.32	1984.07	0.00	398,347.39	0.00
Unbranded	Rock Himalayan Salt	2338.63	0.05	0.10	22.75	3424.70	1.10	0.11	0.00	4519.36	0.00	374,856.60	0.00
Woolworths	Himalayan Pink Fine Table Salt	3431.20	0.82	0.09	132.87	2441.39	2.28	0.00	0.00	2959.88	0.00	408,832.63	0.00
McKenzie’s	Himalayan Pink Rock Salt	4131.17	0.12	0.09	69.63	2513.78	2.44	0.00	0.00	3147.13	0.00	387,043.13	0.00
Woolworths	Pink Himalayan Salt	3266.34	0.08	0.13	41.50	2264.01	1.85	0.00	0.00	3343.51	0.00	392,393.80	0.07

**Table 3:** Composition of non-nutritive minerals in pink salt (*n* = 31) and iodized white table salt (*n* = 1) samples which were purchased from retail stores in Australia in June 2018.

		Non-Nutritive Mineral Content (mg/kg)												
Brand	Product Name	Aluminum	Arsenic	Barium	Boron	Cadmium	Cobalt	Lead	Mercury	Nickel	Silicon	Silver	Sulfur	Vanadium
**Control**														
SAXA	Iodized white table salt	0.00	0.00	0.01	0.00	0.02	0.00	0.02	0.02	0.00	0.000	0.02	431.22	0.00
**Pink salt**														
Affordable wholefoods	Pink Himalayan Salt	30.77	0.00	0.38	0.00	0.00	0.01	0.05	0.01	0.02	0.00	0.00	2491.33	0.00
Coles	Himalayan Pink Salt Grinder	106.29	0.00	0.82	0.00	0.00	0.05	0.05	0.01	0.12	180.07	0.00	6306.48	0.00
Chef’s choice	Himalayan Pink Fine Salt	36.52	0.00	0.72	0.00	0.00	0.03	0.04	0.00	0.11	92.73	0.00	8179.32	0.00
Chef’s choice	Himalayan Pink Rock Salt with Grinder	44.16	0.00	0.94	0.00	0.00	0.04	0.06	0.00	0.00	122.38	0.00	9476.70	0.00
Go Vita	Himalayan Salt	188.34	0.00	1.30	1.08	0.01	0.11	0.04	0.00	0.20	245.39	0.00	5599.26	0.00
Hoyt’s	Pink Himalayan Rock Salt	146.73	0.00	1.28	0.71	0.00	0.07	0.04	0.00	0.14	254.62	0.00	8742.82	0.00
Lotus	Himalayan Salt Coarse	131.54	0.00	1.08	1.13	0.00	0.08	0.06	0.00	0.10	190.77	0.00	5434.24	0.00
Masterfoods	Pink Himalayan Salt	58.33	0.00	0.90	0.00	0.00	0.02	0.07	0.02	0.02	99.14	0.00	7788.20	0.00
McKenzie’s	Himalayan Fine Pink Salt	56.10	0.00	0.46	0.00	0.00	0.03	0.04	0.00	0.05	130.72	0.00	4781.39	0.00
McKenzie’s	Himalayan Pink Rock Salt	101.51	0.00	1.17	0.00	0.01	0.05	0.03	0.02	0.05	213.46	0.00	8703.36	0.00
Mount Zero	Pink lake natural salt	0.00	0.00	0.00	0.87	0.00	0.01	0.00	0.00	0.03	0.00	0.00	11,622.15	0.00
Murray River Gourmet	Salt Flakes Naturally Pink	0.00	0.00	0.06	0.00	0.03	0.00	0.04	0.01	0.14	0.00	0.00	1703.97	0.00
*n* foods	Himalayan Rock Sat	103.14	0.00	0.87	0.00	0.01	0.05	0.03	0.00	0.11	218.18	0.00	7496.16	0.00
Nirvana organics	Himalayan Crystal Salt Medium	80.39	0.00	0.85	0.00	0.00	0.04	0.03	0.01	0.09	126.38	0.00	6922.96	0.00
Nirvana organics	Himalayan Crystal Salt Granules	89.39	0.00	1.02	0.00	0.00	0.05	0.07	0.00	0.10	170.36	0.00	9520.20	0.00
Nirvana organics	Himalayan Crystal Salt Fine	116.11	0.00	0.97	0.74	0.00	0.05	0.05	0.01	0.13	191.29	0.00	4751.24	0.00
Peruvian Pink Salt	Fine Grain Salt	13.68	0.00	0.10	0.00	0.01	0.01	2.59	0.00	0.07	0.00	0.00	3849.69	0.00
Real Food That Loves You Back	Himalayan Pink Rock Salt-Fine	113.23	0.00	0.77	0.00	0.01	0.06	0.05	0.01	0.07	209.02	0.00	5917.59	0.00
Real Food That Loves You Back	Himalayan Rock Salt Crystals	192.65	0.00	1.65	1.15	0.00	0.09	0.07	0.00	0.13	372.10	0.00	10,029.71	0.00
SAXA	Natural Pink Himalayan Table Salt	64.10	0.00	1.12	0.26	0.00	0.03	0.14	0.01	0.25	82.75	0.00	5888.73	0.00
SAXA	Pink Himalayan Salt Flakes	52.28	0.00	0.56	0.00	0.00	0.04	0.12	0.00	0.42	106.05	0.00	4564.46	0.00
SAXA	Natural Pink Himalayan Rock Salt	164.72	0.00	1.34	2.02	0.00	0.09	0.08	0.00	0.16	300.78	0.00	8750.85	0.00
SAXA	Natural Pink Himalayan Salt Grinder	4.24	0.00	0.07	0.00	0.00	0.01	0.03	0.01	0.01	64.01	0.00	4989.18	0.00
SHERU	Fine Himalayan Salt	72.80	0.00	0.74	0.00	0.00	0.03	0.04	0.00	0.11	117.34	0.00	4320.46	0.00
The gluten free co.	Himalayan Salt Fine	15.78	0.00	0.26	0.00	0.00	0.01	0.03	0.00	0.00	33.43	0.00	3722.58	0.00
The gluten free co.	Himalayan Salt Coarse	71.67	0.00	0.85	0.00	0.02	0.04	0.07	0.00	0.02	22.99	0.00	6048.26	0.12
Unbranded	Australian Pink Lake Salt (Medium)	2.26	0.00	0.04	3.04	0.00	0.01	0.00	0.00	0.00	0.00	0.00	33,754.34	0.00
Unbranded	Fine Himalayan Salt	95.05	0.00	0.98	0.00	0.00	0.05	0.04	0.00	0.10	128.09	0.00	4327.64	0.00
Unbranded	Rock Himalayan Salt	35.48	0.00	0.41	0.00	0.00	0.02	0.04	0.00	0.07	16.06	0.00	7120.23	0.00
Woolworths	Himalayan Pink Fine Table Salt	115.28	0.00	1.35	0.00	0.00	0.06	0.09	0.00	0.48	191.10	0.00	6850.12	0.00
Woolworths	Pink Himalayan Salt	61.75	0.00	0.86	0.00	0.00	0.02	0.05	0.01	0.10	181.84	0.00	8032.10	0.00

**Table 4:** Mean nutrient and non-nutritive mineral content per kilogram of pink salt compared to an iodized white salt control.

Minerals	Control ^1^ (*n* = 1)			Pink Salt (*n* = 31)				
		Mean	SD	Range	*p*-Value ^2^	Mean Content in 5 g	Dietary Targets ^4^	Upper Limit
**Nutrients**								
Calcium (mg)	393.28	2695.09	1125.52	530.62–5736.73	<0.001	13.48	1000–1300	2500
Chromium (µg)	0.00	17.48	19.16	0.00–82.09	<0.001	0.87	25–35	-
Copper (mg)	0.08	0.11	0.07	0.01–0.41	0.09	0.00	1.2–1.7	10
Iron (mg)	0.00	63.75	46.89	0.00–167.52	<0.001	0.32	8–18	45
Magnesium (mg)	83.94	2655.31	1956.85	146.78–11,937.98	<0.001	13.28	310–420	350
Manganese (mg)	0.00	2.16	1.49	0.33–8.61	<0.001	0.01	5–5.5	-
Molybdenum (µg)	0.04	1.7	3.7	0.00–15.35	<0.01	0.09	45	2000
Phosphorus (mg)	0.00	1.30	5.35	0.00–28.90	0.19	0.01	1000	4000
Potassium (mg)	151.68	2406.05	1216.52	98.39–4528.89	<0.001	12.03	2800	-
Selenium (µg)	0.00	2.8	6.2	0.00–18.52	0.02	0.14	60–70	400
Sodium (mg)	427,636.27	394,717.96	21,272.20	343,384.79–459,115.58	<0.001	1973.59	460–920 (2000 ^6^)	-
Zinc (mg)	0.00	0.06	0.21	0.00–0.86	0.13	0.00	8–14	40
**Non-nutritive**								
Aluminum (mg)	0.00	76.27	53.90	0.00–192.65	<0.001	0.38	-	30 ^7^
Arsenic (mg) ^5^	0.00	0.00	0.00	0.00–0.00	-	0.00	-	0.5 ^8^
Barium (mg)	0.01	0.77	0.44	0.00–1.65	<0.001	0.00	-	75 ^9^
Boron (mg)	0.00	0.36	0.71	0.00–3.04	<0.01	0.00	-	20 ^10^
Cadmium (mg) ^5^	0.02	0.00 ^3^	0.00	0.00–0.00	<0.001	0.00	-	0.5 ^8^
Cobalt (mg)	0.00	0.04	0.03	0.00–0.11	<0.001	0.00	-	-
Lead (mg) ^5^	0.02	0.13	0.46	0.00–2.59	0.18	0.00	-	0.2 ^8^
Mercury (mg) ^5^	0.02	0.00 ^3^	0.01	0.00–0.02	<0.001	0.00	-	0.01 ^8^
Nickel (mg)	0.00	0.11	0.11	0.00–0.48	<0.001	0.00	-	1 ^10^
Silver (mg)	0.02	0.00	0.00	0.00–0.00	-	0.00	-	-
Silicon (mg)	0.00	131.00	97.22	0.00–372.10	<0.001	0.66	-	-
sulfur (mg)	431.22	7344.70	5413.06	1703.97–33,754.34	<0.001	36.72	-	-
Vanadium (mg)	0.00	0.00 ^3^	0.02	0.00–0.12	0.33	0.00	-	1.8 ^10^

^1^ Iodized, fine white table salt. ^2^ *p*-values derived from 1 sample *t*-test. ^3^ Due to rounding, some values are non-zero. ^4^ Dietary targets are the Australian recommended dietary intake (RDI), except when the RDI is not able to be set and the adequate intake (AI) was used, unless otherwise stated. National Health and Medical Research Council. Nutrient Reference Values for Australia and New Zealand. Canberra, Commonwealth of Australia; 2017. ^5^ Defined as a “metal contaminant Food Standards Australia and New Zealand. Schedule 19: Maximum levels of contaminants and natural toxicants. Canberra, Commonwealth of Australia; 2017. ^6^ Suggested Dietary Target; National Health and Medical Research Council. Nutrient Reference Values for Australia and New Zealand. Canberra, Commonwealth of Australia; 2017. ^7^ Value expressed as mg/kg body weight; FAO/WHO. Evaluation of Certain Food Additives and Contaminants: seventy-fourth report of the Joint FAO/WHO Expert Committee on Food Additives. WHO Technical Report Series 966, 2011. ^8^ Food Standards Australia and New Zealand. Schedule 19: Maximum levels of contaminants and natural toxicants. Canberra, Commonwealth of Australia; 2017. ^9^ Value expressed as mg/kg body weight; Boorman J, Cunningham J, Mackerras D. Salt Intake from Processed Food and Discretionary Use in Australia. Poster presentation. Canberra: Food Standards Australia New Zealand; 2008. ^10^ Institute of Medicine. Dietary Reference Intakes for Vitamin A, Vitamin K, Arsenic, Boron, Chromium, Copper, Iodine, Iron, Manganese, Molybdenum, Nickel, Silicon, Vanadium, and Zinc. The National Academies Press, 2001.

**Table 5:** Impact of origin, form, and color intensity on the mineral content of pink salt samples which were purchased from retail stores in Australia in June 2018.

Minerals	Origin	Form			Color Intensity					
	Himalayas vs. Other Regions	Flakes (3) vs. Fine (1)	Flakes (3) vs. Coarse (2)	Coarse (2) vs. Fine (1)	Dark Pink (3) vs. No Color (0)	Dark Pink (3) vs. Light Pink (1)	Dark Pink (3) vs. Medium Pink (2)	Medium Pink (2) vs. No Color (0)	Medium Pink (2) vs. Light Pink (1)	Light (1) vs. No Color (0)
**Nutrients**	**Mean Difference (95% CI), mg/kg ^1,2^**									
Calcium	817.01	−1092.30	−1714.50	622.10	**2583. 90**	794.10	686.90	1897.00	107.20	1789.80
	(−398.37, 2032.39)	(−3179.10, 994.50)	(−3801.30, 372.30)	(−390.10, 1634.40)	**(572.00, 4595.90)^2^**	(−599.80, 2188.00)	(−398.20, 1772.10)	(−139.20, 3933.20)	(−1321.50, 1535.80)	(−426.40, 4006.00)
Chromium	0.06	0.28	0.32	−0.04	0.15	0.01	−0.11	0.26	0.13	0.13
	(−0.15, 0.28)	(−0.05, 0.62)	(−0.01, 0.66)	(−0.20, 0.12)	(−0.18, 0.47)	(−0.25, 0.28)	(−0.32, 0.09)	(−0.07, 0.58)	(−0.14, 0.40)	(−0.23, 0.50)
Copper	**0.08**	**0.15**	**0.17**	−0.02	0.07	0.00	−0.06	0.13	0.06	0.07
	**(0.00, 0.15) ^1^**	**(0.04, 0.26) ^1^**	**(0.06, 0.28) ^1^**	(−0.07, 0.04)	(−0.07, 0.21)	(−0.09, 0.10)	(−0.13, 0.02)	(−0.01, 0.27)	(−0.04, 0.16)	(0.08, 0.22)
Iron	**54.95**	−23.83	−35.59	11.75	77.25	41.12	9.55	67.69	31.56	36.13
	**(7.04, 102.85) ^1^**	(−113.32, 65.66)	(−125.08, 53.90)	(−31.66, 55.16)	(−13.57, 168.06)	(−21.80, 104.04)	(−39.43, 58.54)	(−24.22, 159.60)	(−32.93, 96.05)	(−63.91, 136.17)
Magnesium	−1982.95	−1577.50	−1879.50	301.90	**−5528.70**	1510.10	352.80	**−5881.60**	1157.20	**−7038.80**
	(−4029.67, 63.77)	(−5279.50, 2124.50)	(−5581.50, 1822.60)	(−1493.80, 2097.70)	**(−8089.40, −2968.00) ^1^**	(−264.00, 3284.20)	(−1028.30, 1734.00)	**(−8473.10, −3290.00) ^1^**	(−661.10, 2975.60)	**(−9859.50, −4218.20) ^1^**
Manganese	−0.62	**3.17**	**2.80**	0.37	1.86	−0.53	0.29	1.57	−0.81	2.38
	(−2.26, 1.03)	**(0.64, 5.69) ^1^**	**(0.28, 5.33) ^1^**	(−0.86, 1.59)	(−1.19, 4.91)	(−2.64, 1.59)	(−1.36, 1.93)	(−1.52, 4.65)	−2.98, 1.35)	(−0.98, 5.74)
Molybdenum	−0.03	0.01	0.01	−0.00	−0.05	−0.04	−0.01	−0.04	−0.03	−0.01
	(−0.07, 0.01)	(−0.06, 0.08)	(−0.06, 0.08)	(−0.04, 0.03)	(−0.12, 0.03)	(−0.09, 0.01)	(−0.05, 0.03)	(−0.11, 0.04)	(−0.08, 0.02)	(−0.09, 0.07)
Phosphorus	−0.81	−2.68	0.00	−2.68	−4.00	−6.44	0.00	−4.00	−6.44	2.45
	(−6.76, 5.15)	(−12.46, 7.10)	(−9.78, 9.78)	(−7.42, 2.06)	(−14.36, 6.36)	(−13.62, 0.73)	(−5.59, 5.59)	(−14.48, 6.49)	(−13.80, 0.91)	(−8.96, 13.86)
Potassium	**2507.87**	−1406.90	−2578.70	**1171.70**	**2747.40**	**1845.50**	533.00	2214.40	1312.50	901.90
	**(1542.99, 3472.74) ^1^**	(−3346.90, 533.00)	(−4518.60, −638.70)	**(230.70, 2112.70) ^1^**	**(836.60, 4658.20) ^1^**	**(521.60, 3169.30) ^1^**	(−497.60, 1563.60)	**(280.60, 4148.20) ^1^**	(−44.40, 2669.30)	(−1202.90, 3006.70)
Selenium	−0.02	0.06	0.08	−0.02	0.01	−0.05	−0.02	0.03	−0.04	0.07
	(−0.09, 0.05)	(−0.05, 0.17)	(−0.03, 0.19)	(−0.07, 0.03)	(−0.11, 0.14)	(−0.14, 0.04)	(−0.09, 0.05)	(−0.10, 0.16)	(−0130, 0.05)	(−0.07, 0.21)
Sodium	4992.71	**60,998.00**	**64,374.00**	−3376.00	30,533.00	−9103.00	−7547.00	38,080.00	−1556.00	39,636.00
	(−18,638.80, 28,624.23)	**(31,763.00, 90,233.00) ^1^**	**(35,138.00, 93,609.00) ^1^**	(−17,557.00, 10,805.00)	(−11,070.00, 72,137.00)	(−37,926.00, 19,721.00)	(−29,986.00, 14,893.00)	(−4025.00, 80,185.00)	(−31,099.00, 27,986.00)	(−6191.00, 85,463.00)
Zinc	**−0.41**	**0.37**	**0.43**	−0.05	0.00	**−0.34**	−0.01	0.01	**−0.32**	0.34
	**(−0.59, −0.24) ^1^**	**(0.03, 0.72) ^1^**	**(0.08, 0.77) ^1^**	(−0.22, 0.11)	(−0.37, 0.37)	**(−0.59, −0.08) ^1^**	(−0.21, 0.19)	(−0.36, 0.39)	**(−0.59, −0.06) ^1^**	(−0.07, 0.74)
**Non-Nutritive Minerals**	**Mean Difference (95% CI), mg/kg ^1,2^**									
Aluminum	**82.99**	−40.55	−61.30	20.75	**98.47**	**68.54**	16.69	81.78	51.85	29.93
	**(31.86, 134.12) ^1^**	(−141.01, 59.91)	(−161.76, 39.91)	(−27.98, 69.48)	**(2.07, 194.86) ^1^**	**(1.76, 135.33) ^1^**	(−35.30, 68.68)	(−15.78, 179.33)	(−16.60, 120.30)	(−76.25, 136.10)
Arsenic ^3^	−	−	−	−	−	−	−	−	−	−
Barium	**0.83**	−0.36	−0.57	0.21	**0.97**	**0.63**	0.14	**0.82**	0.49	0.34
	**(0.45, 1.20) ^1^**	(−1.18, 0.46)	(−1.39, 0.24)	(−0.18, 0.61)	**(0.25, 1.68) ^1^**	**(0.14, 1.12) ^1^**	(−0.24, 0.53)	**(0.10, 1.54) ^1^**	(−0.02, 0.99)	(−0.45, 1.12)
Boron	−0.71	−0.34	−0.39	0.05	**−1.57**	0.39	0.20	**−1.76**	0.19	**−1.95**
	(−1.46, 0.03)	(−1.67, 0.99)	(−1.73, 0.94)	(−0.60, 0.70)	(**−2.77, −0.36) ^1^**	(−0.45, 1.22)	(−0.45, 0.85)	**(−2.98, −0.55) ^1^**	(−0.67, 1.04)	**(−3.28, −0.63) ^1^**
Cadmium	**−0.01**	**0.01**	**0.01**	−0.00	0.00	−0.01	0.00	0.00	−0.01	0.01
	**(−0.02, −0.0) ^1^**	**(0.00, 0.02) ^1^**	**(0.00, 0.03) ^1^**	(−0.01, 0.00)	(−0.01, 0.02)	(−0.02, 0.01)	(−0.01, 0.01)	(−0.01, 0.00)	(−0.02, 0.00)	(−0.01, 0.02)
Cobalt	**0.04**	−0.02	−0.03	0.01	0.04	0.03	0.01	0.04	0.02	0.01
	**(0.01, 0.06) ^1^**	(−0.07, 0.03)	(−0.08, 0.03)	(−0.02, 0.03)	(−0.01, 0.09)	(−0.00, 0.07)	(−0.02, 0.03)	(−0.01, 0.09)	(−0.01, 0.06)	(−0.04, 0.07)
Lead	**−0.60**	−0.14	0.03	−0.17	0.05	−0.50	−0.01	0.06	−0.49	0.55
	**(−1.06, −0.15) ^1^**	(−0.99, 0.71)	(−0.82, 0.88)	(−0.58, 0.25)	(−0.86, 0.97)	(−1.13, 1.14)	(−0.50, 0.48)	(−0.86, 0.99)	(−1.14, 0.16)	(−0.46, 1.56)
Mercury	0.00	0.00	0.00	0.00	0.01	0.00	0.00	0.00	−0.00	0.00
	(−0.01, 0.01)	(−0.01, 0.02)	(−0.01, 0.02)	(−0.01, 0.01)	(−0.01,0.02)	(−0.01, 0.01)	(−0.01, 0.01)	(−0.01, 0.02)	(−0.01, 0.01)	(−0.01, 0.02)
Nickel	0.06	0.17	**0.20**	−0.03	0.07	0.02	−0.10,	0.16	0.11	0.05
	(−0.06, 0.18)	(−0.02, 0.36)	**(0.01, 0.39) ^1^**	(−0.12, 0.06)	(−0.14, 0.27)	(−0.13, 0.16)	(−0.21, 0.01)	(−0.04, 0.37)	(−0.03, 0.26)	(−0.18, 0.28)
Silver ^3^	−	−	−	−	−	−	−	−	−	−
Silicon	**150.41**	−50.19	−107.43	57.24	**179.63**	**147.33**	37.43	142.21	109.90	32.30
	**(58.33, 242.48) ^1^**	(−227.57, 127.18)	(−284.80, 69.94)	(−28.80, 143.27)	**(15.47, 343.80) ^1^**	**(33.59, 261.07) ^1^**	(−51.12, 125.97)	(−23.94, 308.35)	(−6.67, 226.48)	(−148.53, 213.13)
Sulfur	**−6186.04**	−3652.00	−4870.00	1218.00	14,980.00	4489.00	1708.00	**−16,688.00**	2781.00	**−19,469.00**
	**(−11,742.51, −629.56) ^1^**	(−13,893.00, 6590.00)	(−15,112.00, 5372.00)	(−3750.00, 6186.00)	(−21,926.00, −8034.00)	(−323.00, 9301.00)	(−2038.00, 5454.00)	**(−23,717.00, −9658.00) ^1^**	(−2151.00, 7714.00)	**(−27,120.00, −11,818.00) ^1^**
Vanadium	0.00	0.00	−0.01	0.01	0.01	0.01	0.01	0.00	0.00	0.00
	(−0.02, 0.02)	(−0.04, 0.04)	(−0.05, 0.04)	(−0.01, 0.03)	(−0.04,0.05)	(−0.02, 0.04)	(−0.02, 0.03)	(−0.05, 0.05)	(−0.03, 0.03)	(−0.05, 0.05)

^1^*p*-values were derived by Tukey’s post hoc test, and *p* < 0.05 considered statistically significant (shown in bold); ^2^ A positive mean difference indicates a higher nutrient level in samples of Himalayan origin, while a negative mean difference indicates a higher nutrient level in samples from other regions; ^3^ no arsenic or silver (mg/100 g) were detected in any of the pink salt samples.
